# Effectiveness of a 10% imidacloprid/4.5% flumethrin polymer matrix collar in reducing the risk of *Bartonella* spp. infection in privately owned cats

**DOI:** 10.1186/s13071-018-3257-y

**Published:** 2019-02-01

**Authors:** Grazia Greco, Emanuele Brianti, Canio Buonavoglia, Grazia Carelli, Matthias Pollmeier, Bettina Schunack, Giulia Dowgier, Gioia Capelli, Filipe Dantas-Torres, Domenico Otranto

**Affiliations:** 1 Dipartimento di Medicina Veterinaria, Università degli Studi di Bari “Aldo Moro”, Bari, Italy; 2 Dipartimento di Scienze Veterinarie, Università degli Studi di Messina, Messina, Italy; 3 Bayer Animal Health GmbH, Leverkusen, Germany; 4 The Pirbright Institute, Ash Road, Pirbright, Surrey GU24 0NF UK; 5 Instituto Zooprofilattico Sperimentale delle Venezie, Laboratorio di Parassitologia, Legnaro, Italy; 6 Centro de Pesquisas Aggeu Magalhães, Fundação Oswaldo Cruz, Recife, Brazil

**Keywords:** *Bartonella henselae*, *Bartonella clarridgeiae*, Phylogeny, ZF1, Fizz/Cal1, Genotype, Feline VBDs, Incidence, Prevention, Pyrethroids, Flumethrin

## Abstract

**Background:**

*Bartonella henselae*, *Bartonella clarridgeiae* and the rare *Bartonella koehlerae* are zoonotic pathogens, with cats being regarded as the main reservoir hosts. The spread of the infection among cats occurs mainly *via* fleas and specific preventive measures need to be implemented. The effectiveness of a 10% imidacloprid/4.5% flumethrin polymer matrix collar (Seresto®, Bayer Animal Health), registered to prevent flea and tick infestations, in reducing the risk of *Bartonella* spp. infection in privately owned cats, was assessed in a prospective longitudinal study.

**Methods:**

In March-May 2015 [Day 0 (D0)], 204 privately-owned cats from the Aeolian Islands (Sicily) were collared (G1, *n* = 104) or left as controls (G2, *n* = 100). The bacteraemia of *Bartonella* spp. was assessed at enrolment (D0) and study closure (D360) by PCR and DNA sequencing both prior to and after an enrichment step, using *Bartonella* alpha proteobacteria growth medium (BAPGM).

**Results:**

A total of 152 cats completed the study with 3 in G1 and 10 in G2 being positive for *Bartonella* spp. *Bartonella henselae* genotype I ZF1 (1.35%) and genotype II Fizz/Cal-1 (6.76%) as well as *B. clarridgeiae* (5.41%) were detected in cats of G2. *Bartonella clarridgeiae* was the only species detected in G1. Based on the yearly crude incidence of *Bartonella* spp. infection (i.e. 3.85% in G1 and 13.51% in G2; *P* = 0.03) the Seresto® collar achieved a preventative efficacy of 71.54%. The incidence of *Bartonella* spp. infection was more frequent in flea-infested cats (6/33, 18.18%) than in uninfested ones (7/112, 5.88%) (*P* = 0.036).

**Conclusions:**

Cats living in the Aeolian Islands are exposed to *B. henselae* and *B. clarridgeiae*. The Seresto® collar provided significant risk reduction against *Bartonella* spp. infection in outdoor cats under field conditions. Such a preventative tool could be a key contribution for decreasing the risk of *Bartonella* spp. infection in cats and thus ultimately to humans.

## Background

About 35 species of *Bartonella*, small intracellular, fastidious, Gram-negative alpha-proteobacteria, have been described so far [1]. These bacteria are transmitted by a number of vectors, including fleas, and are highly adapted to one or more mammalian hosts, often causing a long-lasting intra-erythrocytic bacteraemia as a hallmark of infection [2, 3]. Cats are the natural reservoir of *Bartonella henselae* and, less commonly, of *Bartonella clarridgeiae* and the rare *Bartonella koehlerae* [4–6].

*Bartonella henselae* exhibits heterogeneity with two serotypes (Houston-1 and Marseille) [7, 8] which overlap genotypes I (Houston I) and genotype II (Marseille), identified according to *16S* rRNA gene sequences [8–11]. In addition, within the above genotypes, two genogroups (Houston I and Marseille II) with more genotypes were delineated based on the heme-binding protein (*Pap31*) encoding gene [10]. The two genogroups above displayed major variations according to animal host species (i.e. humans and domestic cats) and, within the same host species, according to the geographical origin. For example, *B. henselae* genotype II has been predominantly detected in cat populations from western USA, western Europe (France, Germany, Italy, Netherlands, UK) and Australia, whereas genotype I is dominant in Asia (Japan and the Philippines) [12, 13]. The spread of the *Bartonella* spp. infection among cats occurs mainly *via Ctenocephalides felis*, the cat flea [4, 14], in whose digestive system they multiply, surviving several days in the faeces [15, 16]. Indeed, *B. henselae* was experimentally transmitted among cats by transferring fleas fed on naturally infected cats to specific pathogen-free (SPF) cats, and by intradermal injection of excrement collected from fleas fed on *B. henselae*-infected cats [17–20]. Additionally, successful transmission of infection was demonstrated after intradermal injection of blood from cats that had been infected by a virulent field strain of *B. henselae* [20]. Accordingly, possible routes of transmission of *Bartonella* spp. amongst cats include flea bites, contamination of open wounds with excrement of infected fleas, and ingestion of infected fleas or flea faeces [16]. One to three weeks after infection by *B. henselae* cats develop an apparently asymptomatic relapsing bacteraemia, which may persist for months or years [20, 21].

For humans, the main mode of transmission of both *B. henselae* and *B. clarridgeiae* and putatively *B. koehlerae* is *via* contaminated scratches and/or bites of infected cats [22]. Incidental transmission of *B. henselae* to humans can result in a wide range of clinical manifestations also according to the patient’s immune status [23]. In immuno-competent individuals cat-scratch disease (CSD) is characterized by a self-limiting but long-lasting swelling of the lymph node(s) draining the primary site of infection, often associated with high fever and, only rarely, endocarditis, neuroretinitis, uveitis and hepato-splenic abscesses [23–26]. Conversely, immunocompromised patients, such as AIDS patients, may develop bacillary angiomatosis or bacillary peliosis, which are characterized by tumour-like vasoproliferative lesions of the skin or the inner organs, respectively [27]. Similarly, *B. clarridgeiae* is a zoonotic pathogen which may cause asymptomatic haemotropic infection in cats and fever, lymphadenopathy and inoculation papules in humans [28]. Additional, *B. koehlerae* was found associated to culture-negative endocarditis in a human patient [29].

As domestic cats play a central role in the transmission of *Bartonella* to humans, measures for preventing the infection in these pets should be implemented. Healthy *Bartonella* carriers are usually treated with antibiotics [28, 30, 31], but a complete clearance of bacteraemia cannot be guaranteed, presenting the risk of antimicrobial resistance as well as of zoonotic transmission to humans [28, 30, 31]. Consequently, the best approach for preventing *Bartonella* infection in cats relies on the control of flea infestations by using ectoparasitic treatment [32]. A 10% imidacloprid/4.5% flumethrin polymer matrix collar (Seresto®, Bayer Animal Health) with both repellent (anti-feeding) and rapid killing efficacy [33, 34] has been licensed for preventing flea and tick infestations in cats. Studies demonstrated the efficacy of the collar for preventing transmission of vector-borne diseases under experimental and field conditions [35–37]. However, the efficacy against *B. henselae* was exclusively tested under experimental conditions in a restricted number of cats [38] and field studies are lacking. Therefore, the protective effectiveness of the Seresto® collar against feline *Bartonella* spp. infection was assessed in a cohort of privately-owned cats with regular outdoor access, from the Aeolian Islands (Sicily, southern Italy). In that area the occurrence of both *B. henselae* (2.73%, 95% CI: 0.97–4.48), and *B. clarridgeiae* (1.21%, 95% CI: 0.03–2.39) has been reported [39]. Additionally, as more variants of *B. henselae* have been diagnosed in geographical areas where CSD cases have been reported [9, 10, 13], we genetically characterized the population of *B. henselae* in cats involved in this study.

## Methods

### Study site and design

Samples used were collected under the frame of a previous prospective longitudinal, partly blinded, randomized field study aiming at evaluating the protective effectiveness of the collar against *Leishmania infantum* infection in cats [37, 40].

The cohort study was conducted in Lipari and Vulcano, two of the seven islands of the Aeolian archipelago (Tyrrhenian Sea, Sicily, Italy). Briefly, a total of 204 cats belonging to 80 owners were visited and assigned to group 1 (G1, *n* = 104) or group 2 (G2, *n* = 100) following a “per household” assignment random plan in March-May 2015 (D0). The enrolled animals, aged from 6 months to 15 years, were healthy based on clinical examination performed by a veterinarian. The cats in the G1 were treated with the Seresto® collar while those in G2 were left untreated (i.e. no ectoparasiticides were allowed) as controls. Data for fleas were recorded at the enrolment (D0), on D210 (at replacement of collars) and on D360 (study closure) while blood samples were collected on D0 and D360 from the jugular vein. Details on blood sampling as well as ectoparasite collection and processing have been described elsewhere [37, 40, 41]. During the study, cats remained with their owners and were managed as per normal routine without any containment measure or restriction.

### Laboratory procedures

The bacteraemia of *Bartonella* spp. in cats was determined using PCR both prior to and after an enrichment step using *Bartonella* alpha proteobacteria growth medium (BAPGM), and isolation on blood agar slants, as previously described [42]. After thawing, an aliquot of 1 ml of EDTA whole blood was inoculated into 10 ml of BAPGM, after which the cultures were maintained at 35 °C in a 5% CO_2_, water-saturated atmosphere. After a 14-day incubation period, samples were tested by ITS-PCR. A 0.2 ml aliquot of the enrichment BAPGM which tested ITS-PCR positive was inoculated onto blood agar slants and incubated at 35 °C, in a 5% CO_2_, water-saturated atmosphere. Slants were checked for colony formation at 7, 14 and 21 days after inoculation.

Whole genomic DNA for *Bartonella* spp. PCR assays was extracted from EDTA blood as well as BAPGM enrichment liquid blood samples, and from blood agar if colonies were visible. The QIAamp DNA Micro Kit (Qiagen, Milan, Italy) was used according to the manufacturer’s instructions. Reference strains *B. henselae* (PV 252926) and *B. clarridgeiae* (PV85065) were used as positive controls.

*Bartonella* spp. detection was performed using the primers 325s and 1100as designed to amplify the *Bartonella* 16S-23S internal transcribed spacer (ITS) region as described previously [43]. The amplicon size generated is species-dependent, allowing preliminary *B. henselae*, *B. clarridgeiae* and *B. koehlerae* identification.

For *B. henselae* genotyping, all *B. henselae-*positive samples underwent a second step PCR targeting the *Pap-31* gene by using the species-specific primers Pap31-BHSs and Pap31-688as, according to previously reported methodology [43]. The PCR assays were performed using a DNA Thermal Cycler Gene AMP 9600 (Applied Biosystem, Milan, Italy) in a 25 μl final volume containing 2 μl of DNA, 12.5 ml of AccuPrimeTM SuperMixII mix (Invitrogen, Milan, Italy) (40 mM Tris-HCl pH 8.4, 3 mM MgCl_2_, 100 mM KCl, 400 mM of each dNTP, AccuPrimeTM Taq DNA Polymerase), 200 pM of each primer and DNase-free H_2_O up to 25 μl. PCR products were examined on 2% agarose gels stained with GelRed (VWR International PBI, Milano, Italy) and visualized on a GelLogic 100 gel documentation system (Kodak, New York, USA).

All of the generated ITS and *Pap31* PCR products were sequenced in both directions using the same primers as for PCR by Eurofins Genomics (Vimodrone, Italy) for *B. clarridgeiae*/*B. koehlerae* species identification and for *B. henselae* genotyping, respectively. The newly-generated sequences were edited, aligned and compared to reference GenBank sequences by nucleotide BLASTN program (https://blast.ncbi.nlm.nih.gov). Phylogenetic analyses were carried out using the software package Geneious version 10.1.3 (Biomatters Ltd., Auckland, New Zealand). For phylogenetic tree construction, a 400-nt fragment of the *B. henselae Pap31* gene was analysed using Geneious Tree Builder. The Neighbor-Joining method was used with data resampled 1000 times to estimate the confidence of branching patterns. Representative sequences obtained in the study were submitted to the GenBank database.

Additionally, cats were also screened for feline haemoplasma bacteraemia. DNA of three feline haemoplasma species, *Mycoplasma haemofelis* (*Mhf*), “*Candidatus* Mycopasma haemominutum” (*C*Mhm) and “*Candidatus* Mycoplasma turicensis” (CMt), were molecularly detected in blood samples by three specific real-time PCR assays as previously described [44]. Real-time PCR was performed in a 25-μl reaction mixture containing 12.5 μl of iTaqTM Universal Probes Supermix (Bio-Rad Laboratories Srl, Segrate, Milan, Italy), 600 nM of primers, 200 nM of probe and 10 μl of DNA. For each assay, positive controls included DNA extracted from blood samples of cats naturally infected with each haemoplasma species (laboratory collection). The thermal protocol consisted of activation of iTaq DNA polymerase at 95 °C for 10 min, followed by 45 cycles of denaturation at 95 °C for 15 s, annealing at 48 °C for 30 s and extension at 60 °C for 1 min.

### Data management and statistical analyses

A minimum sample size of 70 cats was estimated for each group, based on the assumptions of confidence level of 95%, power of 80% and expected incidence of *Bartonella* spp. infection of 1% and 13% in treated and untreated cats, respectively. A cat was considered infected with *Bartonella* spp. if it tested positive in at least one of the diagnostic tests employed (PCR on EDTA blood, PCR on BAPGM and on blood agar colonies). The year-crude incidence (YCI) of infected/infested cats in each group on D360 was calculated as follows:

$$ \mathrm{YCI}=\left(\mathrm{No}.\mathrm{of}\ \mathrm{infected}\ \mathrm{animals}/\mathrm{No}.\mathrm{of}\ \mathrm{negative}\ \mathrm{animals}\ \mathrm{included}-\mathrm{No}.\mathrm{of}\ \mathrm{animals}\ \mathrm{not}\ \mathrm{completing}\ \mathrm{the}\ \mathrm{study}\right)\times 100 $$

The efficacy in preventing *Bartonella* spp. infection and flea infestation was based on YCI and calculated as follows:

$$ \%\mathrm{protection}=\left(\mathrm{YCI}\ \mathrm{in}\ \mathrm{untreated}\ \mathrm{group}-\mathrm{YCI}\ \mathrm{in}\ \mathrm{treated}\ \mathrm{group}/\mathrm{YCI}\ \mathrm{in}\ \mathrm{untreated}\ \mathrm{group}\right)\times 100 $$

The differences between YCI in G1 and G2 were tested for statistical significance using Fisher’s exact test or Chi-square test, where appropriate.

Associations between variables (age, sex, flea infestation, lymph node enlargement, *Leishmania infantum* infection, haemotropic *Mycoplasma*) and *Bartonella* spp. infection were statistical analysed using a Chi-square or Fisher’s exact test, where appropriate. Relative risk (RR) at a 95% confidence interval (CI) was used to determine the magnitude of associations. The software used was WinEpi [45] and the statistical significance threshold for two-tailed tests was set at *P* < 0.05.

## Results

Of the 204 cats enrolled, 152 animals (78 from G1 and 74 from G2) were available for assessment of *Bartonella* spp. whereas the remaining animals were excluded for different reasons (Table 1). Amongst the excluded cats, 13 (seven from G1 and six from G2) were removed after the enrolment because they were identified as already infected with *Bartonella* spp. at inclusion on D0. The two groups of cats included in the analysis were not statistically different for sex (*χ*^2^ = 0.696, *df* = 1, *P* = 0.4) and age (*χ*^2^ = 0.008, *df* = 1, *P* = 0.93) at the time of inclusion (D0) (Table 1).

**Table 1:** Number and characteristics of cats treated with the Seresto® collar (G1) and untreated controls (G2) that either completed or were excluded from the study

	G1	G2	Total
Completed the study			
No. of cats	78	74	152
Median age in months (interquartile range)	24 (10–48)	18 (10–36)	18 (10–36)
Sex *n* (%) female/male	39/39 (50)	42 (56.8)/32 (43.2)	81 (52.29)/71 (46.71)
Excluded from the study			
Number of cats	26	26	52
Infected with *B. henselae* at the inclusion	2	5	7
Infected with *B. clarridgeiae* at the inclusion	5	1	6
Deceased^a^	6	9	15
Suspected adverse drug reaction	1	–	1
Lost to follow up	12	11	23

^a^Car trauma (*n* = 4); suspected infectious disease (*n* = 3); respiratory failure (*n* = 1); aortic thromboembolism (*n* = 1)

**Fig. 1:**
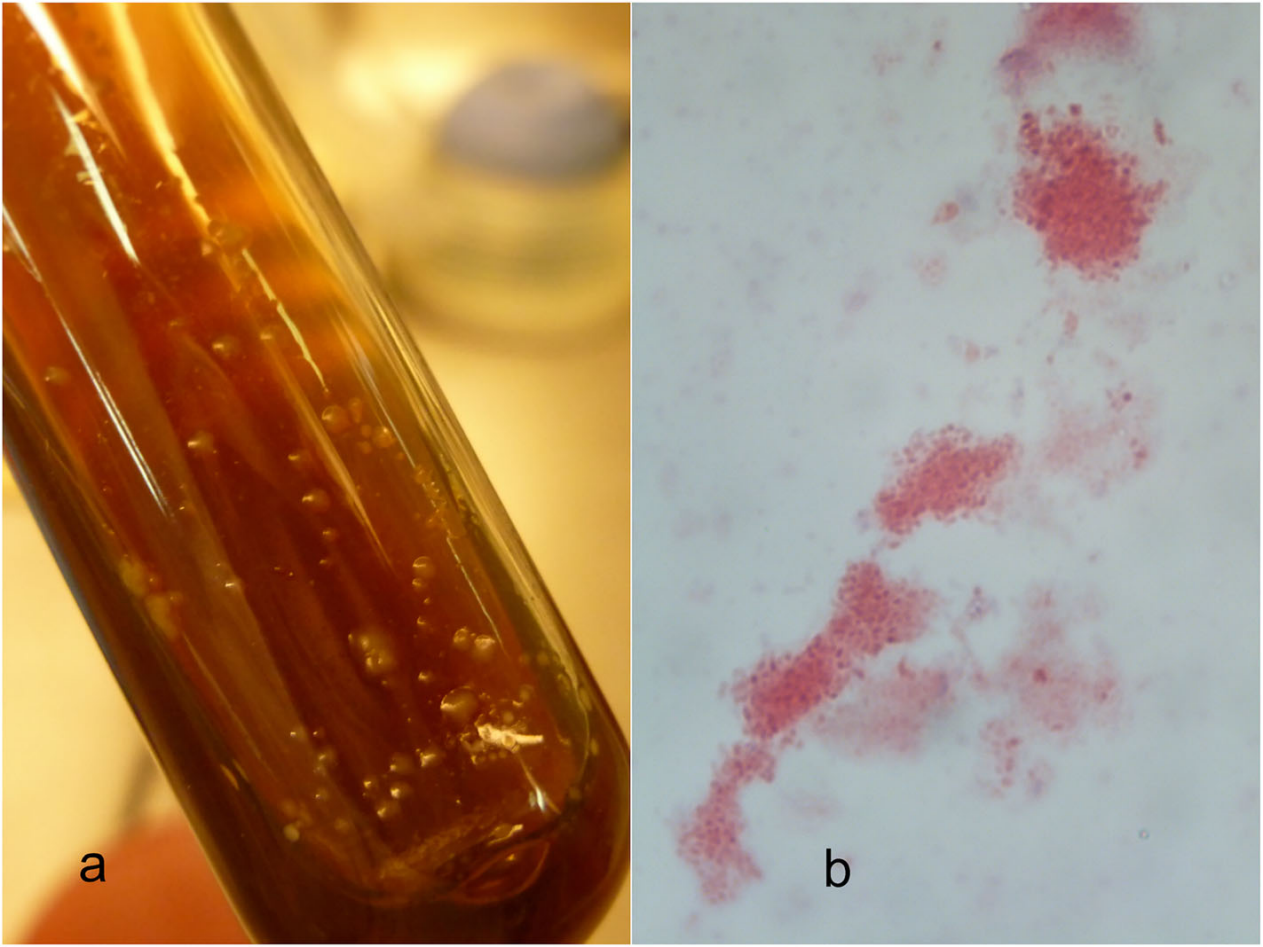
**a** Growth on blood agar slant (Oxoid®) supplemented with 5% defibrinated sheep blood. First-passage 21-day-old LipBh_4 isolate of *B. henselae* genogroup II, genotype Fizz/Cal1. Slants had been kept at 35 °C in a 5% CO_2_ atmosphere. *B. henselae* appears as circular brownish colonies, with humid aspect, sticking to the agar. **b** Light microscopy images (100×) of Gimenez-stained LipBh_4 isolate of *B. henselae* genotype II

On samples collected on the final study day (D360), ITS-PCR assays performed on both DNA extracted from EDTA blood samples and after BAPGM enrichment step yielded identical results. Out of 78 cats from G1 and 74 cats from G2 tested, 3 (3.85%) and 10 (13.51%) were found positive, respectively; among those from G2, 3 were also found positive by blood agar slant sub-cultures (Table 2, Fig. 1). Using ITS and *Pap31* gene combined PCR assays, 3 out of 78 cats (3.85%) in G1 and 4 out of 74 cats (5.41%) in G2 were infected by *B. clarridgeiae* and 6 out of 74 cats (8.11%) in G2 scored positive to *B. henselae* (Table 2). Out of three cats infected by *B. clarridgeiae* in G1 two (# G1_43 and G1_76, Table 2) were found infested by fleas at D 210*.*

**Table 2:** Results of PCR from both whole blood samples and after BAPGM enrichment and from isolates on blood agar for *B. henselae* and *B. clarridgeiae* in cats treated with the Seresto® collar (G1) or in untreated controls (G2) after being exposed to one transmission season in a highly endemic area (D360)

Group (*n*)	*Bartonella* spp., *n* (%)^a^	*B. clarridgeiae, n* (%)^b^	*B. henselae*, *n* (%)^c^	*B. henselae* isolates, *n* (%)^d^
G1 (78)	3/78 (3.85)	3/78 (3.85)	0	0/3
G2 (74)	10/74 (13.51)	4/74 (5.41)	6/74 (8.11)	3/10
Total (152)	13/152 (8.55)	7/152 (4.6)^e^	6/152 (3.9)^f^	3/13 (23)^g^

^a^ITS PCR from whole blood and enrichment BAPGM

^b^ITS sequence analysis

^c^*Pap*31 PCR

^d^*B. henselae* isolated on agar blood culture after enrichment BAPGM step

^e^Sample IDs: G1_43, G1_56, G1_76, G2_132, G2_147, G2_172 and G2_173

^f^Sample IDs: G2_27, G2_60, G2_72, G2_90, G2_106 and G2_149

^g^Sample IDs: G2_60, G2_90 and G2_149

**Table 3:** Reference and field strains of *Bartonella* spp*.* used for sequence and phylogenetic analyses

*Bartonella* species	*16S* genogroup/serotype	*Pap*31 genotype	Strain	Sample ID	GenBank ID^a^	Reference (R) / Field strains (F)
*B. henselae*	I	Houston I	Houston I	–	AF001274	R
*B. henselae*	I	PV262926	PV_1	–	ns	R
*B. henselae*	I	ZF1	60457	–	AF321116	R
*B. henselae*	I	ZF1	LipBh_3	G2_27	MH350808	F
*B. henselae*	II	Marseille	Marseille	–	AF308169	R
*B. henselae*	II	Fizz		–	AF308167	R
*B. henselae*	II	Fizz/Cal1	LipBh_4	G2_60	MH350809	F
*B. henselae*	II	Fizz/Cal1	LipBh_5	G2_72	MH350810	F
*B. henselae*	II	Fizz/Cal1	LipBh_6	G2_90	MH350811	F
*B. henselae*	II	Fizz/Cal1	LipBh_7	G2_106	MH350812	F
*B. henselae*	II	Fizz/Cal1	LipBh_8	G2_149	MH350813	F
*B. clarridgeiae*	16S-23S ITS		–	–	AF167989	R
*B. clarridgeiae*	16S-23S ITS		LipBc_1	G1_43	MH348146	F
*B. clarridgeiae*	16S-23S ITS		LipBc_2	G2_132	MH348147	F

^a^MH350808–13 and MH348146–7 sequences were generated in this study (F)

*Abbreviation*: *ns* not submitted to GenBank

All seven partial ITS *B. clarridgeiae* sequences obtained in the study showed 100% homology to sequences of *B. clarridgeiae* reference strain AF167989. Due to the 100% identity of the sequences obtained in this study only two representative sequences were deposited in the GenBank database with the corresponding accession numbers reported in Table 3.

**Fig. 2:**
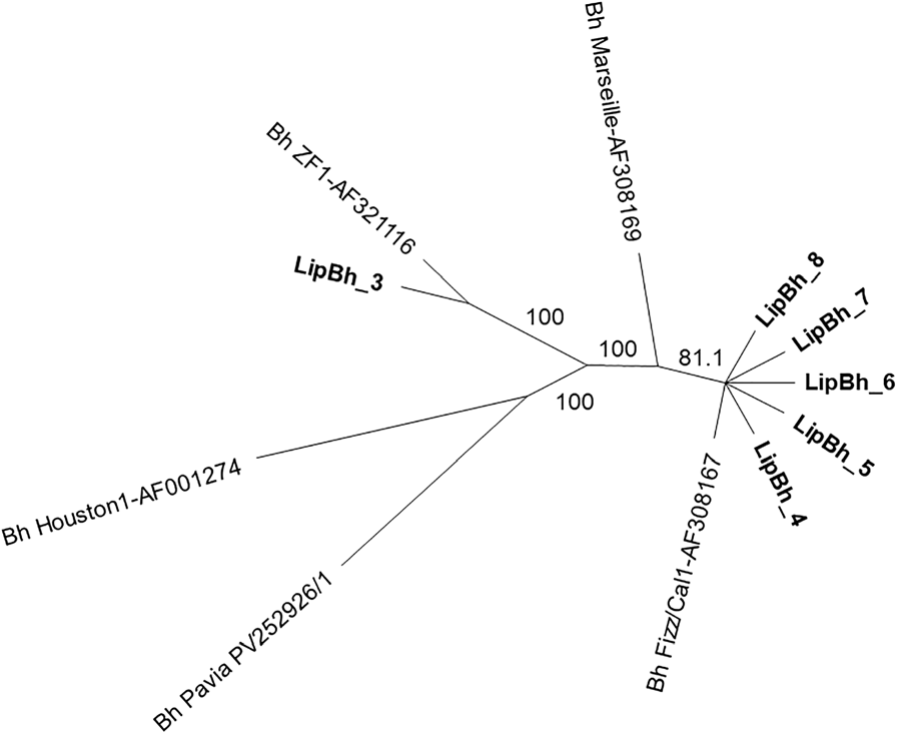
Neighbor-joining unrooted tree (obtained by using the Geneious® 10.3.1 software package, Biomatters Ltd, Aukland, New Zealand) generated with *pap31*-based sequencing data for 5 reference and 6 field *B. henselae* variants. The numbers at the nodes indicate the percentages of occurrence of the branching order in 100 bootstrapped trees for the neighbour-joining trees. The strains generated in this study are indicated in bold. All field strains were from the cats of the G2 group (unprotected control animals). The *B. henselae* strain LipBh_3 (GenBank: MH350808) detected from the cat G2_27 was 99.77% identical to the genotype Bh ZF1 (GenBank: AF321116), inside genogroup I. Strains LipBh_4 to LipBh_8 (GenBank: MH350809-MH350813) isolated from the cats G2_60, _72, _90, _106 and *_*149 clustered into the genotype Fizz/Cal1 (GenBank: AF308167) of the genogroup II Marseille

Four *B. henselae* reference strains, one for each known *Pap-31* genotype, were included in the analyses (Table 3). BLAST search and nucleotide alignment with *B. henselae* reference sequences showed that *B. henselae* strains detected in this study belonged to *Pap31* genogroup I (*n* = 1) and genogroup II (*n* = 5) (Fig. 2, Table 3). The percentages of nucleotide identity of the sequences obtained in this study with the closest reference strains available in the GenBank database were as follows: LipBh_3, 99.77% nucleotide identity with *B. henselae* ZF1 (AF321116) strain of genogroup I; LipBh_4 to _8 strains, 99.77% nucleotide identity with strain Fizz/Cal1 (AF308167) of the *B. henselae* genogroup II. Five *B. henselae* genogroup II strains (LipBh_4 to _8) displayed a 100% nucleotide identity with each other.

The analysis confirmed the subtyping obtained by sequence analysis. *Bartonella henselae* genotype I ZF1 (*n* = 1, 1.35%) and *B. henselae* genotype II Fizz/Cal-1 (*n* = 5, 6.76%) were detected in cats from G2.

Both *B. henselae-* and *B. clarridgeiae-*infected cats were evaluated together in a risk factor analysis. *Bartonella* spp. infection was more frequent in cats younger than 12 months-old than in elder ones (11.86 *vs* 6.65%; *χ*^2^ = 1.35, *df* = 1, *P* = 0.25) and equally distributed between male and female cats (9.86 *vs* 7.41%; *χ*^2^ = 0.29, *df* = 1, *P* = 0.59). The YCI was 3.85% in G1 and 13.51% in G2 (*P* = 0.03) leading to 71.54% efficacy of the collar in preventing *Bartonella* spp. infection.

Of the 152 cats analysed for *Bartonella* spp*.*, 126 animals (67 from G1 and 59 from G2) were available for assessment of haemotropic *Mycoplasma* incidence whereas the 26 remaining were excluded from the analysis because they were identified to be already infected with haemotropic *Mycoplasma* at inclusion on D0. Out of 67 cats from G1 and 59 cats from G2 tested, 7 (10.45%) and 8 (13.56%) were found haemotropic *Mycoplasma* spp.-positive, with *C*Mhm being more prevalent; among those from G2, two were co-infected with *B. henselae* or *B. clarridgeiae*. Only one cat in G2 was infected with *M. haemofelis*. No significant difference of incidence (*χ*^2^ = 0.29, *df* = 1, *P* = 0.59) for *Mycoplasma* spp. was detected between the two groups.

At enrolment (D0), G1 and G2 had a comparable percentage of cats infested by fleas (24.35 *vs* 27.02%; *χ*^2^ = 0.142, *df* = 1, *P* = 0.71) (Table 4). During the course of the study, the percentage of flea infested cats in G1 was reduced to 4 cats (5.1%, *χ*^2^ = 19.638, *df* = 1, *P* = 0.0001) at D210 and none (0%, *χ*^2^ = 42.266, *df* = 1, *P* = 0.0001) at D360 (Table 4). Conversely, for G2, flea infestation was observed in 24 (32.4%) cats at D210 and 33 (44.6%) cats at D360, with no significant prevalence variation in the infestation rates (*χ*^2^ = 5.295, *df* = 3, *P* = 0.0708). This resulted in efficacies against fleas of 84.26 and 100% on D210 and D360, respectively. At the study closure all cats were healthy based on clinical examination; however, 11 (14.10%) cats in G1 and 28 (37.84%) in G2 showed peripheral lymph node enlargement being more frequent in animals of the G2 group than in those of G1 (*χ*^2^ = 11.21, *df* = 1, *P* = 0.0008). Of the 152 cats included in the analysis three (3.85%) in G1 and 20 (23.03%) in G2 tested *Leishmania* spp. positive, as reported in a previous study [37].

**Table 4:** Number and percentages of flea infested cats from treated (G1) or untreated (G2) groups, at the enrolment (D0), during treatment (D210) and at the end of experiment (D360) with Seresto® collar

Study day (D)	D0		D210		D360	
Group	G1 (*n* = 78)	G2 (*n* = 74)	G1 (*n* = 78)	G2 (*n* = 74)	G1 (*n* = 78)	G2 (*n* = 74)
Flea infestation, *n* (%)	19 (24.35)	20 (27.02)	4 (5.1)^a^	24 (32.4)^a^	0^b^	33 (44.59)^b^

Significant differences are marked with equal letters

^a^*χ*^2^ = 18.839, *df* = 1, *P* < 0.0001

^b^*χ*^2^ = 44.430, *df* =1, *P* < 0.0001

While no significant association was detected between *Bartonella* spp. and *Leishmania infantum* (*χ*^2^ = 0.001, *df* = 1, *P* = 0.98) or *Bartonella* spp. and peripheral lymph node enlargement (*χ*^2^ = 3.131, *df* = 1, *P* = 0.08), significant association was recorded between *Leishmania infantum* and peripheral lymph node enlargement (*χ*^2^ =13.53, *df* = 1, *P* = 0.0002; RR = 2.8, 95% CI: 1.62–4.87).

## Discussion

Cats of the Aeolian Islands are exposed to *B. clarridgeiae*, *B. henselae* and haemotropic *Mycoplasma*. The Seresto® collar proved to be effective in reducing the risk of *Bartonella* infection in cats under natural field conditions with an overall efficacy of 71.54%.

The PCR on whole blood samples prior to and after BAPGM enrichment step, blood agar slant culture and DNA sequencing approach used in this study detected *B. clarridgeiae* and variants of *B. henselae* infections in the feline population examined. Both bacterial culture and PCR amplification from blood were used as elective diagnosis supporting an infection at the time of sampling [46], instrumental to the assessment of the prevention efficacy of the collar against *Bartonella* spp. infection in cats.

In our study 13 out 152 (8.55%) blood samples tested positive, thus revealing the *Bartonella* spp. infection in cats from the Aeolian Islands. No differences were observed for yield of *Bartonella* spp. detection by using PCR assay after BAPGM enrichment step compared with PCR results obtained from whole blood samples without enrichment, according to a previous study [47]. Additionally, BAPGM enrichment step did not improve the isolation rate, with only 3 out of 13 (23%) PCR positive samples yielding growth of *Bartonella* spp. colonies on blood agar slants. Several factors may affect the isolation of *Bartonella* spp. in culture, including the species of *Bartonella*, the magnitude and the duration of bacteraemia and the maintenance of organism viability from the sample collection time to its cultivation [48]. The higher number of BAPGM enrichment-PCR positive samples (*n* = 13) as compared with agar slant sub-cultures (*n* = 3) may indicate both the amplification of non-viable bacteria in blood samples that failed to grow in solid cultures and the difficulty in cultivating and isolating the *Bartonella* spp. from reservoir and non-reservoir patients [42]. Therefore, PCR assay on ITS of blood may be the best approach for screening population animals, being faster than BAPGM enrichment liquid culture which takes at least four weeks to yield definitive results [48].

The overall incidence of *Bartonella* spp. was 8.55% (13/152), being 3.85% (3/78) in treated animals compared to 13.51% (10/74) in untreated control animals. Of the two species, *B. henselae* was more frequent (6/74, 8.11%) than *B. clarridgeia*e (4/74, 5.41%) in the G2 group kept under natural conditions. This is similar to data found in a recent study in the area and in different European countries [49–53].

Two main pathogenic *B. henselae* variants belonging to Houston I and Marseille II genogroups have been identified in both animals and humans, and both of these variants are involved in CSD. *B. henselae* genogroup II-genotype Fizz/Cal-1 was predominant in Sicily being detected with the highest frequency (6.76%) in G2 cats and similar to findings in cats from north Italy [12], France [17] and Germany [23]. Comparatively, only one cat in G2 harboured the less frequent *B. henselae* genogroup I-genotype ZF1.

Although no significant association was observed between incidence and sex (*P* = 0.25) or age (*P* = 0.08) of the cats examined, cats younger than one year-old showed a higher prevalence for *Bartonella* spp. than elder ones, as already demonstrated in the USA and the Netherlands [9]. These data further support the hypothesis that juvenile cats, rather than adults, may be more efficient reservoirs of CSD for humans with an association between owning a kitten and CSD [22]. Clinical signs were not related to *Bartonella* spp. infection (*P* = 0.08) according to previous studies [54], whereas cats infected with *Leishmania infantum* were nearly three times more at risk to develop peripheral lymph node enlargement (*χ*^2^ = 13.53, *df* = 1, *P* = 0.0002; RR = 2.8, 95% CI: 1.62–4.87).

Haemoplasma infection is common in cats worldwide [49] and these infections are recognized in Italy [55]. In the present study, 11.9% of the cats were positive for *Mycoplasma* spp., with *C*Mhm being the most prevalent species, similar to previous studies [53]. Although it is still unknown how feline hemoplasmas are transmitted, vector transmission through fleas [56] or ticks [57] has been suggested. In the present study, no difference in incidence (*χ*^2^ = 0.29, *df* = 1, *P* = 0.59) was recorded in cats treated with the Seresto® collar (G1) or in untreated controls (G2) after being exposed to one transmission season in highly endemic area (D360) thus supporting a major role of direct transmission (e.g. aggressive interactions) among cats.

By virtue of its anti-flea activity the Seresto® collar significantly decreased the incidence of *Bartonella* spp. infection (*χ*^2^ = 4.54, *df* = 1, *P* = 0.03; RR = 3.51, 95% CI: 1.0062–12.69) in cats with an overall efficacy of 71.54%. Cats included in this trial were at high risk of *Bartonella* infection with the study being carried out in a highly endemic area for *Bartonella* spp. [36, 55, 58]. The vast majority of cats lived constantly outdoors in suburban or rural areas, with a lifestyle at high risk of flea infestation, as previously reported [40]. The collar was efficacious in controlling flea infestation in the population of cats (*χ*^2^ = 29.027, *df* = 3, *P* < 0.0001), thus reducing the risk of infection in collared cats as compared to non-collared cats. This result mirrors a previous study performed in Japan [59] where a significantly higher seroprevalence of *B. henselae* was observed in cats with flea infestations than that in flea-free cats, thus highlighting the main role of fleas for the infection under natural conditions.

The YCI of *B. clarridgeiae* (i.e. 3.85%) in cats negative for fleas in the collared cats only apparently conflicts with the epidemiological and pathogenic mechanisms of *Bartonella* spp. infection in cats, which are strictly related to the contact with fleas or their excrement [17–20]. Indeed, two of the three collared cats infected by *B. clarridgeiae*, which were older than two years at the enrolment (D0), had already been found infested by fleas at D210 [37], although we cannot state the role of those fleas as we did not test *Bartonella* spp. infection either in fleas or cats at D210. Furthermore, we cannot exclude that those three cats were already infected with *B. clarridgeiae* at enrolment. Indeed, after the infection exposure, bacteria colonize the vascular endothelial cells (primary niche) and the bacteraemia only begins four to five days later [2]. Accordingly, those cats could have tested negative as the level of bacteraemia may have been at low level at D0 and thus undetectable, or they had only just been infected.

## Conclusions

Cats living in the Aeolian Islands are exposed to *B. henselae*, *B. clarridgeiae*, haemotropic *Mycoplasma* and *Leishmania infantum*. The Seresto® collar represents a useful preventive measure against infections with both *B. henselae* and *B. clarridgeiae*, in addition to *Leishmania infantum* [40], therefore providing individual protection for cats living in *Bartonella* endemic areas that could potentially act as reservoirs of the pathogen to humans.

## Abbreviations

AIDS: Acquired immune deficiency syndrome
BAPGM: *Bartonella* alpha proteobacteria growth medium
CSD: Cat scratch disease
D: Study day
ITS: Internal transcribed spacer
RR: Relative risk
YCI: Year crude incidence
